# Clinical features of prostate cancer by polygenic risk score

**DOI:** 10.1007/s10689-024-00369-0

**Published:** 2024-04-15

**Authors:** Christina Spears, Menglin Xu, Abigail Shoben, Shawn Dason, Amanda Ewart Toland, Lindsey Byrne

**Affiliations:** 1 Division of Human Genetics, Department of Internal Medicine, College of Medicine, The Ohio State University, 2012 Kenny Road, Columbus, OH 43212 USA; 2 Comprehensive Cancer Center, The Ohio State University, Columbus, OH USA; 3 Department of Internal Medicine, College of Medicine, The Ohio State University, Columbus, OH USA; 4 Division of Biostatistics, College of Public Health, The Ohio State University, Columbus, OH USA; 5 Division of Urologic Oncology, The Ohio State University Comprehensive Cancer Center, Columbus, OH USA; 6 Department of Cancer Biology and Genetics, College of Medicine, The Ohio State University, Columbus, OH USA

**Keywords:** Polygenic risk score, Prostate cancer, Genetic counseling, Genetic testing

## Abstract

Genome-wide association studies have identified more than 290 single nucleotide variants (SNVs) associated with prostate cancer. These SNVs can be combined to generate a Polygenic Risk Score (PRS), which estimates an individual’s risk to develop prostate cancer. Identifying individuals at higher risk for prostate cancer using PRS could allow for personalized screening recommendations, improve current screening tools, and potentially result in improved survival rates, but more research is needed before incorporating them into clinical use. Our study aimed to investigate associations between PRS and clinical factors in affected individuals, including age of diagnosis, metastases, histology, International Society of Urological Pathology (ISUP) Grade Group (GG) and family history of prostate cancer, while taking into account germline genetic testing in known prostate cancer related genes. To evaluate the relationship between these clinical factors and PRS, a quantitative retrospective chart review of 250 individuals of European ancestry diagnosed with prostate cancer who received genetic counseling services at The Ohio State University’s Genitourinary Cancer Genetics Clinic and a 72-SNV PRS through Ambry Genetics, was performed. We found significant associations between higher PRS and younger age of diagnosis (p = 0.002), lower frequency of metastases (p = 0.006), and having a first-degree relative diagnosed with prostate cancer (p = 0.024). We did not observe significant associations between PRS and ISUP GG, histology or a having a second-degree relative with prostate cancer. These findings provide insights into features associated with higher PRS, but larger multi-ancestral studies using PRS that are informative across populations are needed to understand its clinical utility.

## Introduction

One in eight individuals with a prostate in the United States will be diagnosed with prostate cancer in their lifetime [1]. Despite this high prevalence, the genetic etiology of prostate cancer remains largely unknown. Currently, there are 14 moderate to high penetrance genes associated with hereditary prostate cancer (*ATM, BRCA1, BRCA2, CHEK2, EPCAM, HOXB13, MLH1, MSH2, MSH6, NBN, PALB2, PMS2, RAD51D,* and *TP53*), conferring a 2- to 6- fold increased lifetime risk; however, only 14% of individuals diagnosed with prostate cancer will have a pathogenic variant in one of these genes, suggesting there are additional genetic and environmental factors contributing to development of this disease [2, 3]. Genome-wide association studies (GWAS) have identified more than 290 single nucleotide variants (SNVs) associated with prostate cancer [4]. Most cancer associated SNVs confer very small increases in risk (< 1.2 odds ratios [ORs]) and individually offer little predictive value; however, their combined effects are estimated to account for 33% of the familial risk of prostate cancer [5]. Polygenic Risk Scores (PRS) are a measurement of the combined effect of inherited risk variants. They are generated by a weighed sum of the risk associated with each variant and the number of alleles each individual has inherited using effect sizes identified from GWAS [6].

PRS may have clinical utility by improving predictive values of screening tools such as serum prostate-specific antigen (PSA) and digital rectal exams (DRE); studies suggest that individuals with a high PSA and PRS may benefit most from further diagnostic testing [7, 8]. The first prospective clinical trial evaluating the use of a PRS in prostate cancer screening is the BARCODE1 study [7]. Using a 130-SNV PRS, twenty-five individuals had a PRS in the top 10% and were invited to undergo MRI and biopsy [7]. Of those eligible, nine out of 20 had an abnormal MRI (45%) while 18 underwent biopsy and 7 were diagnosed with prostate cancer (38.8%) [7]. All 7 prostate cancers were low-risk with a mean PSA of 1.8 ng/mL, which could have been missed with PSA alone [7]. The results from this clinical trial suggest that PRS could be used to stratify individuals at a higher risk to develop prostate cancer, while individuals with a lower PRS could potentially avoid unnecessary screening and diagnostic procedures. Another prospective study followed more than 10,000 men in the United States over a period of 20 years and utilized a 279-SNV PRS in addition to family history to identify individuals at highest risk of dying from prostate cancer before age 75 [8]. This study indicates PRS with family history, could potentially be used to decrease morbidity and mortality by identifying individuals at the highest risk for prostate cancer [8]. One Finnish-population based retrospective study comparing individuals with and without prostate cancer using a 55 SNV PRS, found a significantly higher percentage of individuals had a PSA level of ≥ 4 ng/mL in the highest PRS quartile compared to the lowest quartile (18.7% vs 8.3%, P-value < 0.00001) suggesting that adding the PRS to PSA testing could contribute additional information in predicting prostate cancer risk [9]. The study also found an association between high PRS and metastatic disease; however, no association between Gleason score or advanced stage was found [9].

PRS may further stratify an individuals’ risk of developing prostate cancer through incorporation of additional clinical factors, but studies to assess the associations are limited. The AmbryScore™ PRS is one such risk tool that provides an estimate of prostate cancer risk for both affected and unaffected individuals based on patient-specific factors such as age at testing, ethnicity, results of germline genetic testing and results of SNV profiling [5]. The AmbryScore™ includes 72 prostate cancer associated SNVs, is weighted by the SNV-specific effects reported in large prostate cancer studies, and includes ethnicity-specific allele frequencies for which it was validated [5]. Scores are normalized to 1; individuals with scores less than 1.0 are considered to have a lower genetic risk for developing prostate cancer while individuals with a score above a 1.0 are considered to have a higher genetic risk for prostate cancer development [5].

Although numerous studies have assessed clinical and other features of individuals in prostate cancer GWAS and PRS generation research studies, studies of clinically ascertained individuals are lacking. To address this gap, this study aimed to investigate how International Society of Urological Pathology (ISUP) Grade Group (GG), histology, age of diagnosis and family history correlate with PRS amongst individuals with prostate cancer who do not have a germline pathogenic variant in a known prostate cancer related gene and were ascertained in a clinical setting.

## Methods

A quantitative retrospective chart review of 250 individuals diagnosed with prostate cancer who received genetic counseling services between January, 2019 – September, 2022 at the Ohio State University’s Genitourinary Cancer Genetics Clinic, and an AmbryScore™ PRS was conducted. Participants were included in the study if they self-reported their ancestry as Non-Hispanic White and were diagnosed with prostate cancer up to age 84, due to AmbryScore™ only being validated in Whites of less than 85 years of age. To receive an AmbryScore™, the study participants must have tested germline negative (not carry a pathogenic or likely pathogenic variant) for the 14 known prostate cancer genes. They may have a variant of unknown significance (VUS) in these genes.

### Chart review categorization

All clinical features regarding participants prostate cancer were abstracted from pathology reports.

- Family history: The family history was self-reported by participants during their genetic counseling session and was defined as having of one or more first- or second-degree relatives diagnosed with prostate cancer at any age. First and second-degree family histories were assessed separately.
- PRS: PRS categories were defined as low, intermediate, and high. A PRS of 1.0 is the population average. We considered PRS from 0.8 to 1.2 as intermediate or average. The low PRS category included PRS of less than 0.8 and the high PRS category included PRS of greater than 1.2.
- ISUP GG: ISUP GG was separated into three categories. The low-grade group included GG 1. The intermediate grade group included GG 2–3 while the high-grade group included GG 4–5.
- Metastases: Metastatic cancer was defined as regional nodal or distant metastases.
- Age at diagnosis: Age of diagnoses was defined as a continuous variable as well as a categorical variable. The categorical variable involved two categories: individuals diagnosed at the age of 60 and younger and individuals diagnosed at the age of 61 and older.
- Histology: The histology of the individual’s prostate cancer was categorized by the presence versus absence of ductal or intraductal prostate cancer**.**

### Data analysis

Descriptive statistics were used to quantify the distribution of PRS, both as a continuous value and as well as grouping into clinically relevant categories: < 0.8 (low), 0.8–1.2 (average), and > 1.2 (high). Clinical features were then compared across category of risk score using Chi-squared tests. In the case of sparse cell sizes, Fisher’s exact test was used instead to avoid anti-conservative inference. A total of six associations were formally tested with the overall type I error rate controlled at 0.05 using the Holm correction for multiple comparisons.

## Results

A total of 292 charts were reviewed resulting in 250 individuals meeting study inclusion criteria as detailed above. Of the 250 individuals, 67 (27%) had a low PRS of 0.8 or lower, 72 (29%) had an average PRS between 0.8–1.2 and 111 (44%) had a high PRS of 1.2 or greater (Table 1). We discovered a relationship between higher PRS and younger age of diagnosis (p = 0.002), lower frequency of metastases (p = 0.006), and increased likelihood of having a family history of a first-degree relative diagnosed with prostate cancer (p = 0.024). We did not observe a statistically significant relationship between PRS and ISUP GG (p = 0.27), histology (p = 0.29), or having a second-degree relative diagnosed with prostate cancer (p = 0.68).

**Table 1:** Clinical features of prostate cancer by PRS

	PRS low (< 0.8) n = 67	PRS average (0.8–1.2) n = 72	PRS high (> 1.2) n = 111	p value
Age 61–80	39 (58%)	39 (54%)	38 (34%)	0.002^a^
Age 39–60	28 (42%)	33 (46%)	73 (66%)	
ISUP GG				
Low (1)	4 (6%)	12 (15%)	13 (11%)	0.27^b^
Medium (2–3)	21 (31%)	25 (32%)	41 (41%)	
High (4–5)	42 (63%)	41 (53%)	57 (57%)	
Metastases (reginal nodal or distant)	41 (61%)	31 (43%)	41 (37%)	0.006^a^
Non-metastases	26 (39%)	41 (57%)	70 (63%)	
Intraductal/ductal histology presence	8 (12%)	9 (13%)	7 (6%)	0.29^b^
Intraductal/ductal absence	59 (88%)	63 (88%)	104 (94%)	
*First-degree relative +	23 (34%)	33 (46%)	61 (55%)	0.024^a^
*First-degree relative −	44 (66%)	38 (54%)	49 (45%)	
*Second-degree relative	14 (21%)	18 (25%)	22 (20%)	0.68^a^
*Second-degree relative −	53 (79%)	53 (75%)	88 (80%)	

_*Sample size 248 due to two unknown family histories_

_+Participant reported having at least one first or second degree relative diagnosed with prostate cancer_

_−Participant did not report having a first or second degree relative diagnosed with prostate cancer_

^a^_Chi-square test_

^b^_Fisher’s exact test_

The majority of individuals with a high PRS score were diagnosed at age 60 or younger (66%), while most individuals with a low PRS score were diagnosed over age 60 (58%). Additionally, 63% of individuals with a high PRS score did not show evidence of metastatic disease, while 61% of individuals with a low PRS score had evidence of metastatic disease. Finally, 55% of individuals with a high PRS score, had a family history of prostate cancer in a first-degree relative while 66% of individuals with a low PRS score did not have a first-degree relative with prostate cancer.

## Discussion

### Age

Increased age is the most common risk factor for prostate cancer with 67 being the median age of diagnosis in the United States [1, 10, 11]. Our study revealed a significant inverse association between the age of prostate cancer diagnosis and PRS (p = 0.002). Other studies that investigated associations of PRS with age, but did not account for germline genetic testing, found that individuals with a low PRS (< 1% of risk) were an average of 65 years of age at diagnosis, while those with a high PRS (≥ 99%) were diagnosed at an average of 56 years of age [12]. In our study, 26 individuals (10%) were diagnosed in their 40’s, and this age group accounts for less than 7% of the general population of individuals diagnosed with prostate cancer [11]. Thus, our study had a higher proportion of younger individuals than the general population. Of those individuals, 15 (58%) had a high PRS, six (23%) had an average PRS and five (19%) had a low PRS.

Not all study participants with high PRS were diagnosed at younger ages. Reasons for this may be due to other genetic and lifestyle factors that can influence the age of prostate cancer diagnosis. For example, our oldest participant was diagnosed at age 80 and had a PRS of 1.4, putting this individual into the high PRS category. This individual had a high ISUP GG of 5 and a son and brother diagnosed with prostate cancer at age 77 and 53 respectively. This individual may have had protective factors that delayed his diagnosis despite his high PRS and strong family history. Interestingly, the participant with the highest PRS score in our study (PRS = 5) was diagnosed with prostate cancer at age 71. This participant had a high ISUP GG of 5 and a father with prostate cancer diagnosed in his 70s. This participant also has a p.E546G VUS in *MSH6*, a known prostate cancer gene, which is still classified as a VUS at the time of the study. It is possible that the 72-SNV PRS used for our study overestimated the genetic risk in these two individuals and a larger PRS (e.g. 290 known SNVs) would have resulted in a lower risk score. These results highlight the need for additional research to fully understand lifestyle, sociodemographic, and genetic factors contributing to prostate cancer development.

### ISUP GG

Similar to other studies that have investigated the association between PRS and ISUP GG, we did not identify a statistically significant association between ISUP GG and the PRS (p = 0.29) [9, 13]. Interestingly, most individuals in all three PRS categories fell into the high ISUP GG. A reason for this unexpected finding could be due to individual characteristics of who was referred to a genetic counselor. National Comprehensive Cancer Network (NCCN) guidelines recommend genetic testing to all individuals who have an ISUP GG of 4 or 5 [14]. Of the 250 patients 131 of them (52%) had an ISUP GG of 4 or 5. As such, we would expect more individuals with high ISUP GG scores to be seen in our clinic. It’s also possible there are SNVs associated with ISUP GG that weren’t included on the AmbryScore™ or have not yet been identified through GWAS.

### Metastases

Our data revealed a statistically significant inverse association between PRS and whether the prostate cancer metastasized to local nodal or distant region (p = 0.006). Other studies that have investigated this relationship, but which did not account for germline genetic testing, have found that individuals with high PRS have a higher incidence of metastatic disease, which is opposite of the relationship our study discovered [9, 15]. In the general population, the vast majority (77%) of prostate cancer cases are localized, while approximately 13% of prostate tumors have spread to regional lymph nodes, and 6% have distant metastasis upon diagnosis [16]. Our study had a higher proportion of metastasis observed in the prostate cancer population as a whole. In our cohort, 45% of participants had evidence of metastatic disease either to a lymph node or to bone, while 55% remained localized. Reason for the differences between our study population and the general population is likely due to the nature of participants that are referred to see a genetic counselor as NCCN recommends genetic testing for all individuals who have been diagnosed with metastatic disease [14]. It is possible that if all individuals with prostate cancer seen clinically were given a PRS that our inverse association may have disappeared or reversed.

### Family history

Our study discovered a statistically significant association between a high PRS and having a first-degree relative diagnosed with prostate cancer (p = 0.024) but no association with having a second-degree relative (p = 0.68). Other studies that have investigated the association between PRS and family history of prostate cancer without accounting for germline genetic testing have also found that incorporating family history of prostate cancer improves the predictive value of PRS [8]. Family members share genetic variants and therefore, may be more likely to have similar PRS scores although PRS do not capture all of the known genetic risk for disease. In our study, the highest number of family members diagnosed with prostate cancer in one family was seven: the proband diagnosed at age 62, his father (55), brother (68), paternal uncle (68), and three paternal cousins diagnosed at age 60, 62, and 68. The individual had a PRS score of 0.8 and fell into the low PRS category. Reasons for the individual with the strongest family history having a low PRS could be that our proband did not carry a pathogenic variant in a high-risk gene that was in other members of this individual’s family. Alternatively, this individual could have a pathogenic variant in a gene that isn’t included on Ambry’s cancer panel or has yet to be discovered. It’s possible that some of the major contributing risk SNPs for this individual were not included on Ambry’s PRS as 72 of the known 290 SNVs associated with prostate are included.

Other studies that have investigated the association without accounting for germline genetic testing, did not find that incorporating family history improved the predictive value of PRS [17]. Additional research that has investigated family history of other common diseases and PRS supports family history and PRS can be complementary variable but can also be independent variables [18]. Because the family history was relayed by patient knowledge and memory, it may not be accurate. Individuals may not be aware of other family members diagnosis if they are not in close contact or if individuals in their family don’t share their medical history. Individuals may mistake a different type of cancer such as bladder or testicular cancer as prostate cancer. Therefore, there could be a higher or lower incidence of family history than was reported. Additionally, there can be environmental and lifestyle factors that can contribute to someone’s prostate cancer diagnosis.

### Study limitations

This study has some limitations. There are other contributing risk factors associated with prostate cancer and PRS not evaluated in this study. Importantly, diet, lifestyle and socioeconomic factors may contribute to a prostate cancer development [1]. Individuals in this study may have had a pathogenic variant in a high-risk cancer-associated gene outside the 14 studied here which may have affected their risk. Another limitation of this study is that the AmbryScore™ includes only 72 of the more than 290 known prostate cancer related SNVs and is only available to individuals of European ancestry due to limited predictive value in other populations. As GWAS studies have only discovered approximately 10–30% of SNVs associated with their prospective cancer [19], it is likely that there are many more prostate cancer risk variants. Finally, this study is a single institution study of a very specific prostate cancer cohort consisting of only 0.1% of the ~ 248,530 individuals diagnosed with prostate cancer in the United States each year [11]. As such, results may not be representative or generalizable of the entire population or those from other institutions. Furthermore, our study population is largely dependent upon referrals from physicians and only considers individuals who have been referred and agree to meet with a genetic counselor. The prostate cancer genetic counseling referral guidelines given to physicians at The Ohio Stat University include being diagnosed with prostate cancer and having one of the following: diagnosed under age 55, having Ashkenazi Jewish ancestry, metastatic disease, ISUP GG 4 or 5, or a family history of prostate, breast, ovarian, pancreatic, colorectal, kidney, urothelial, and/or endometrial cancer in one or more family member. Having specific referral criteria as outlined, can lead to ascertainment bias. Additionally, is difficult to know how many individuals were offered genetic counseling and declined, or whose physician simply did not present them with the option of genetic counseling. It is possible that individuals who decline genetic counseling or did not get offered the genetic testing due to not meeting the referral guidelines, may have different characteristics compared to those included in this study.

## Conclusions

Significant associations between PRS and age of diagnosis, metastatic disease, and family history of a first-degree relative with prostate cancer was identified in our study. However, PRS cannot predict if or when an individual may develop prostate cancer as there are multiple factors not included in this study that can contribute to prostate cancer development and diagnosis. To our knowledge, no other studies have investigated the relationship between PRS and clinical factors in individuals with prostate cancer in a clinically ascertained group, while taking into account negative germline genetic testing of known prostate-cancer related genes.

In order to determine if PRS can have clinical utility, PRS in individuals with and without prostate cancer needs to be evaluated through additional randomized clinical trials and offered to more than just those of European ancestry. To increase predictive value, PRS should include all the SNVs and environmental and clinical risk factors that have been linked to prostate cancer. Additionally, identifying SNVs associated with higher grade prostate cancers could decrease morbidity and mortality of individuals with prostate cancer. Understanding the associations of PRS and prostate cancer clinical characteristics in affected individuals could help in defining the clinical utility of PRS in screening individuals with and without a prostate cancer diagnosis and potentially help with morbidity and mortality for those at high risk.
